# Opportunities and obstacles to the development of nanopharmaceuticals for human use

**DOI:** 10.1186/s40199-016-0163-8

**Published:** 2016-10-06

**Authors:** Nasser Nassiri Koopaei, Mohammad Abdollahi

**Affiliations:** 1 Department of Pharmaceutics, School of Pharmacy, University of Florida, Orlando, USA; 2 Department of Toxicology & Pharmacology, Faculty of Pharmacy and Pharmaceutical Sciences Research Center, Tehran University of Medical Sciences, Tehran, Iran; 3 Endocrinology and Metabolism Research Center, Endocrinology and Metabolism Clinical Sciences Institute, Tehran University of Medical Sciences, Tehran, Iran; 4 Toxicology Interest Group, Universal Scientific Education and Research Network, Tehran University of Medical Sciences, Tehran, Iran

**Keywords:** Human Use, Nanopharmaceuticals, Toxicity Profile, Regulatory Framework

## Abstract

**Abstract:**

Pharmaceutical nanotechnology has generated breakthrough developments in improving health care and human life from its emergence. The biomaterials employed mainly aim at improving drug delivery systems, imaging and diagnostic technologies while the nanoscale materials are in widespread use in other industries such as electronics and optics. Such advancement may revolutionize the drug development and therapy with new and more efficient treatments. Although, nanotechnology assists humankind in improving its well being, it has certain limitations that entail thorough investigation by the regulatory and scientific authorities. To address concerns regarding the safety and toxicity profile of the nanopharmaceuticals, we have reviewed the challenges and solutions of nanopharmaceuticals use in human health and the related health risks. In this regard, regulatory and scientific bodies such as countries’ Food and Drug Administration (FDA), Organization for Economic Co-operation and Development (OECD), European Medicine Agency (EMA), Environmental Protection Agency (EPA), National Institute for Occupational Safety and Health (NIOSH), and World Health Organization (WHO) can participate in developing and reinforcing safety measures and regulatory frameworks to insure the public health. The regulatory authorities may enforce the nanopharmaceutical industries to conduct comprehensive toxicity tests and monitor the adverse drug reaction reports in close collaboration with the scientific community to act accordingly and inform the public as the implementation of the strategy.

**Graphical abstract:**

Nanopharmaceuticals have tremendous potential for human use as therapeutic or diagnostic agents. But their toxicity profile should be well addressed and the respective regulatory framework developed and reinforced by the authorities.
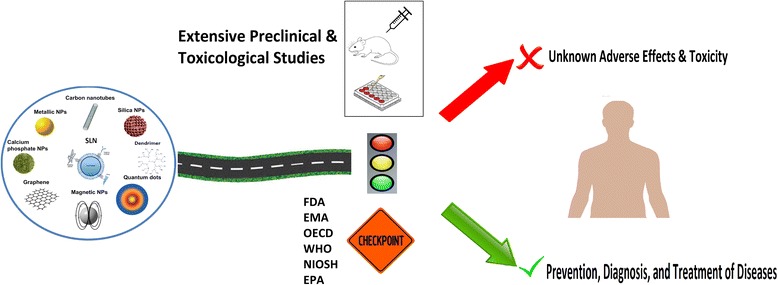

## Introduction

Pharmaceutical nanotechnology deals with the scope of pharmaceutical compounds that emerge from a multidisciplinary field of science entailing the development and application of molecular structures with dimensions smaller than 100 nm [1]. The nanoscale feature of these products and devices render them special capabilities that can be used to produce devices and products as therapeutic agents and other purposes [1, 2]. Pharmaceutical nanotechnology has generated breakthrough developments in health care and human life [3]. The biomaterials employed in this field mainly aim at improving drug delivery systems, imaging and diagnostic technologies while the nanoscale materials are of widespread use in other industries such as electronics and optics [4]. In 1995 with the approval of Doxil® (liposomal doxorubicin) by the US FDA, the pharmaceutical nanotechnology passed its first evolutionary milestone with the subsequent approval of other products but the approvals are in their early stage with less than 50 US FDA approved drug formulations while more are in the pipeline [1, 5, 6]. However, nanotechnology applications in the treatment of some of most critical metabolic and genetic diseases and cancer, delivery systems, genetic tests, as well as imaging and diagnostics are promising enough to absorb huge amounts of investment into research and development efforts both in the academia and the industry [1, 4, 5]. Noteworthy, nanotechnology provides humankind with exceptional opportunities to improve its wellbeing, but it also has certain limitations that entail thorough investigation by the regulatory and scientific authorities [6, 7]. The present study reviews the literature on the pharmaceutical application of nanomaterials and then raises the question regarding the possible health risks associated with nanopharmaceuticals use in humans. Concerns about human use of nanomaterials become overwhelming when we know that these nanostructures do not completely abide by the scientific principles that form the basis for our knowledge about how human physiologic system deals with exogenous compounds and toxicity tests also fail to adequately address the nanotoxicity issues for human beings, animals and the environment. Moreover, the research strives to bring the toxicological aspect of nanopharmaceuticals and pending health risks to top agenda in regulatory bodies as well as scientific community.

## Nanopharmaceuticals: advantageous applications and toxicological concerns

Liposomes, niosomes, polymer based micelles, nanostructured vaccines, polymersomes, dendrimeric nanostructures are nanoparticulate structures used as novel and targeted drug delivery systems. These nanostructures have special structural and functional capabilities and aspects that offers specific applications when engineered like the composition and percentage of components, size distribution and physical structure. In the sense, the nanoparticles could be responsive to the environment, targeted or assume other specificities [8, 9].

**Fig. 1:**
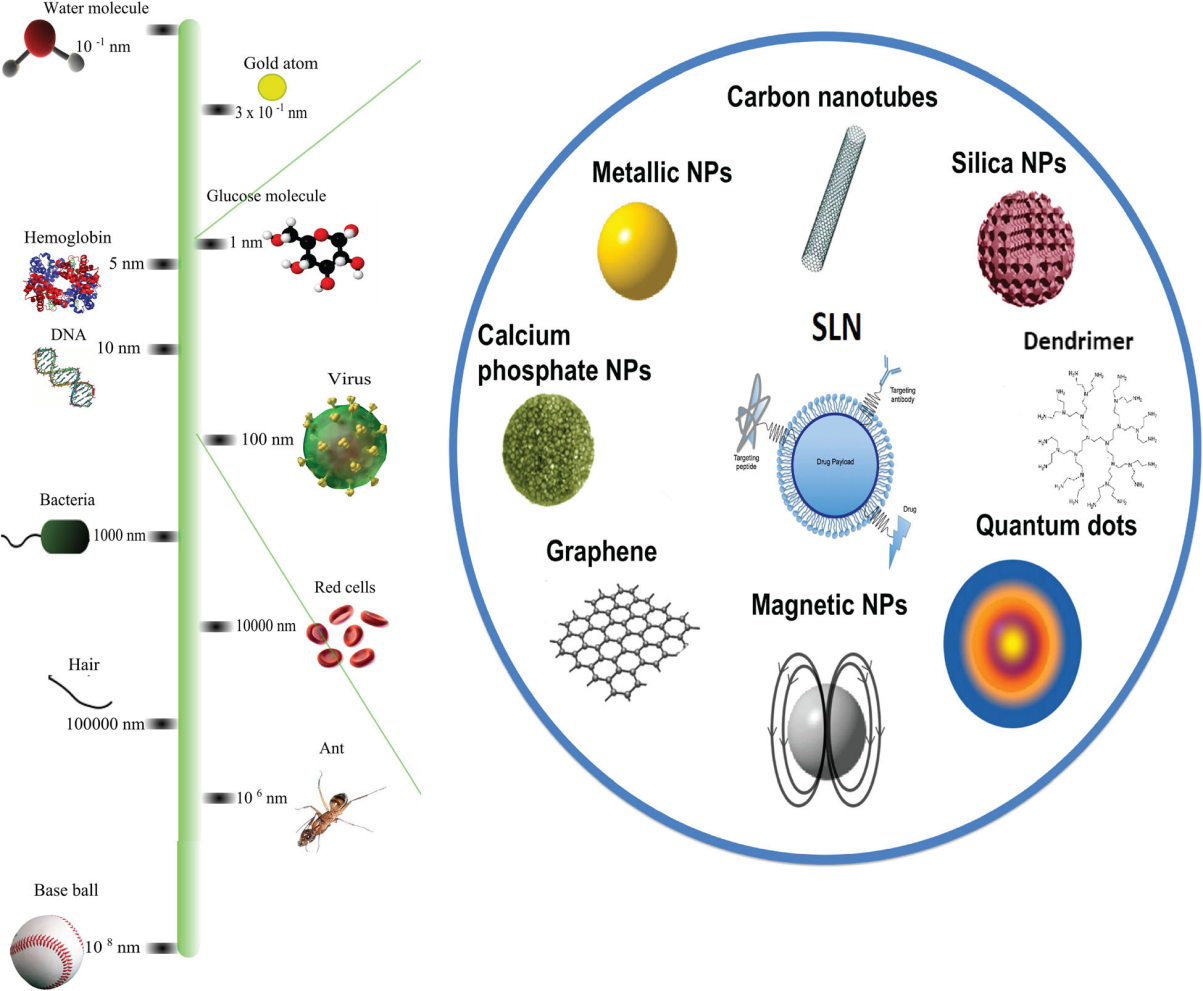
Schematic representations of some nanomaterials with pharmaceutical applications (by AuSbj (Own work) [CC BY-SA 4.0 (http://creativecommons.org/licenses/by-sa/4.0) (https://commons.wikimedia.org/wiki/File%3ANanomaterials_enhanced_SPR.png)], via Wikimedia Commons) within the micro and macro size range (by Sureshbup (http://www.mdpi.com/1422-0067/15/5/7158) [CC BY-SA 3.0 (http://creativecommons.org/licenses/by-sa/3.0)], via Wikimedia Commons and SLN by Andrea Trementozzi (Own work) [CC BY-SA 3.0 (http://creativecommons.org/licenses/by-sa/3.0) (https://commons.wikimedia.org/wiki/File%3ASolidLipidNanoparticle.jpg)], via Wikimedia Commons)

**Fig. 2:**
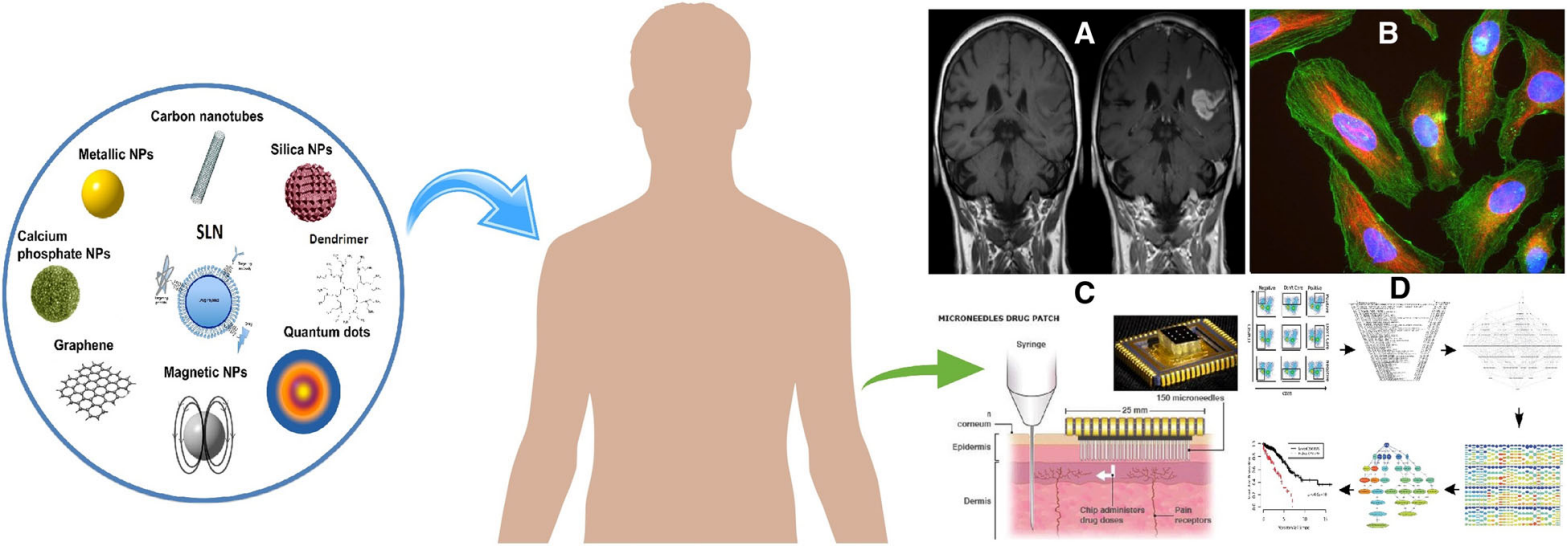
Applications of nanomaterials as (A) MRI contrast agents adapted from ﻿Wikimedia Commons [by Hellerhoff (own work); CC BY-SA 3.0 (http://creativecommons.org/licenses/by-sa/3.0) (https://commons.wikimedia.org/wiki/File%3ABluthirnschranke_nach_Infarkt_nativ_und_KM.png)], via Wikimedia Commons), (B) drug delivery targeting using antibody (by Gerry Shaw [GFDL (http://www.gnu.org/copyleft/fdl.html) or CC BY-SA 3.0 (http://creativecommons.org/licenses/by-sa/3.0) (https://commons.wikimedia.org/wiki/File%3AHeLa_cells_stained_with_antibody_to_actin_(green)_%2C_vimentin_(red)_and_DNA_(blue).jpg)], via Wikimedia Commons), (C) novel drug delivery devices (by National Health Federation [CC BY-SA 3.0 (http://creativecommons.org/licenses/by-sa/3.0) (https://commons.wikimedia.org/wiki/File%3ATransdermal_microneedles.png)], via Wikimedia Commons) and (D) treatment optimization by developing marker based cell characterization (by Nima Aghaeepour et al. [Public domain], via Wikimedia Commons (https://commons.wikimedia.org/wiki/File%3AFlowType-RchyOptimyx.png))

Special characteristics of nanostructures make them useful agents for both diagnosis and imaging agents (Fig. 1) [10]. Nanochips and nanoarrays employ different methods to measure various biomarkers within biologic samples to monitor disease formation and progression. Moreover, nanotheranostics contain both diagnostic and pharmacotherapeutic agents in one formulation to attain various purposes. They may be aimed at drug delivery, drug release, drug efficacy and therapeutic drug monitoring. In addition, different nanoparticles have been developed to improve the diagnostic capabilities of nuclear magnetic resonance imaging (Fig. 2) [11, 12].

With all the features in mind, there are certain concerns with regards to nanoparticulate structure for human use [13]. Nanoparticles toxicity has attracted the most vital criticism because they represent exceptional characteristics such as size, size distribution, surface charge and properties, expanded surface area, self-assembly and stability [4].

These features influence the nanoparticles’ ADME (Adsorption, Distribution, Metabolism and Excretion) properties like cellular uptake, distribution within the body fluids, and transport through biological barriers. For instance, the tiny molecular size of the nanoparticles enables them to cross the natural biological barriers in the brain and eye or other cells. Route of administration also determines the toxicity profile to a lesser extent as for example, nanopharmaceuticals may trigger neurotoxicity and inflammatory responses or even the systemic circulation when applied in inhalation forms through their penetration into the CNS via posterior nasal mucosal layer [14]. However, our knowledge is yet scant enough not to be sure about the mechanistic toxicology of nanoparticles since the currently available toxicity tests are not fully assuring and data obtained from in vitro or animal studies are not always extrapolatable to human [15, 16].

Exposure to nanopharmaceuticals may affect manufacturing personnel, healthcare professionals and the patients. However, when the nanopharmaceuticals are disposed or excreted via waste water, the general public will also be at exposure. Therefore, elaborate monitoring systems based on physicochemical characterization are required [1, 15]. As the nanoparticles enter the body, they penetrate the epithelial and endothelial barriers and then undergo cellular uptake processes like diffusion, different endocytosis pathways such as receptor mediated endocytosis dictated by their physicochemical and surface properties. Then their biodistribution also follows relatively unknown patterns rather than those of other conventional pharmaceuticals, though such organ deposition in the Central Nervous System (CNS) and growing fetus would be of dramatic concern along with other organs like liver, kidney and spleen. Although nanopharmaceuticals cytotoxicity mechanisms are not well defined, but oxidative stress, proinflammatory effects and genotoxicity are theocratized via Reactive Oxygen Species (ROS) formation, GSH/GSSG ratio alteration, upregulation of transcription factors and signaling kinases, DNA damage and mutation [1, 17]. Cytotoxicity and genotoxicity have been seen with nanodrugs containing metallic ions like aluminum oxide, gold, copper oxide, silver, zinc oxide, titanium oxide, iron oxide and carbon based nanomaterials. Although silica and polymer based nanomaterials have been deemed to be more biocompatible and relatively nontoxic, they may also involve ROS formation and cytotoxicity [9]. Some toxicity tests have been proposed for the nanopharmaceuticals before their approval both in vitro and in vivo. In vitro tests may include but not limited to uptake and transport characterization, cytotoxicity assays (Lactate Dehydrogenase, tetrazolium, Alamar Blue, OECD developed Neutral Red dye assays that are based upon Neutral Red dye cellular uptake), genotoxicity (Ames test, comet assay, micronucleus and chromosomal aberration assays) and carcinogenotoxicity (SHE test, BALB/c 3 T3 and C3H10T1/2 assays). Validated in vivo tests should include at least ADME studies and acute and chronic organ toxicity tests; however, these in vivo tests are time and cost consuming and entail ethical compromises [1, 9, 18]. OECD guidelines provide a wealth of technical documents for the toxicity testing of nanomaterials, but the OECD is yet developing the standard materials critical for the testing, working on the development of carcinogenotoxicity tests like SHE test, BALB/c 3 T3 and C3H10T1/2 assays. The OECD guideline for the tests on routine chemicals are useful in this regard to some extent, but they need more detailed discussion on the biohazards and physicochemical characterizations [1].

## Regulatory framework for nanopharmaceuticals

Concerns regarding the safety and toxicity profile of the nanopharmaceuticals involve active participation of regulatory and scientific bodies, including but not limited to FDA, EMA, EPA, NIOSH, WHO to develop and reinforce safety measures and regulatory frameworks to insure the public health [7, 19].

In this venue, scientific associations as well as the scientific committees within regulatory authorities like US FDA and EMA release technical documents and recommendations for the industry and regulators that lead mostly the evaluation and approval of the products and devices as per the postulation of the public health and quality of life. US FDA has recently updated its regulatory approach to the nanopharmaceuticals through guidelines and denoted that for products that contain nanoscale materials or properties attributable to the dimensions, may require premarket review or where not applicable, urges the industry to consult the FDA very early in the product development phase to address the product’s regulatory status and concerns with regards to its safety, effectiveness or public health impact [20]. Moreover, the FDA declared its regulatory policy towards the nanopharmaceuticals earlier. It stated that the body would be scientifically focused on the product filing and applies for premarket review or consultation approach as per the rules and regulations. The FDA already keeps the industry responsible for its products meet all the legal requirements like safety, effectiveness and other product quality attributes. Nevertheless, the authority continues to run post-market monitoring surveys to protect the consumers while maintaining its role in preparing technical documents and advisory guidelines [21]. The bioequivalent versions of the nanotechnology derived products or nanosimilars compose a new era of extensive regulatory burden. In addition, regulatory authorities of other countries have to keep in pace with the market trend and mobilize their capacities for the oncoming nanosimilars. Overall, the nanoparticulate biomaterials such as liposomes and dendrimers involve more elaborate investigations caused by their modified formulations and ADME [3, 22].

Nanopharmaceuticals approval should contain close characterization of physicochemical properties as they have size and size distribution that may increase the chance of thromboembolic complications via facilitating the thrombosis cascade. On the other hand, nanopharmaceuticals require sophisticated stability and ADME studies because most of the critical parameters of their safety and efficacy may evade during shelf life and cause morbidities and fatalities. Moreover, FDA has approved some liposomal, dendrimer based, PEGylated and albumin bound compounds while the overwhelming number of candidates are on their way to market [23–26].

It should not be forgotten that discovery of new medicines has been always expensive, time-consuming, competitive, with unknown outcome to pass all preclinical and clinical phases of approval. Therefore, to reduce the attrition rate in further steps, investigators must pay enough attention to safety of medicines, development risks, dose ranging, early proof of concept/principle, and patient stratification based on biologically and/or clinically validated biomarkers [27].

## Future prospects of nanopharmaceuticals in human health

With the advent of nanosized structures and their special characteristics, many fields of science started to take advantage of these extraordinarily modifiable and functional features based on their special needs. Medical and pharmaceutical experts also employed these capabilities to develop new drugs, medical devices and therapeutic methods. Cancer is a major cause of mortality and morbidity that has benefited from nanopharmaceuticals and promising progress is also on the way. In spite of the currently approved drugs for cancer chemotherapy, there are more than twenty candidates in clinical trials for approval process. These candidates either improve therapeutic/toxicity profile of existing drugs or contain novel molecules [28, 29]. Dermatology is also applying nanostructures for different diseases treatment because of the special structure of skin and natural barriers that could be simply overcome through noninvasive methods using the nanosized structures [30]. Drug delivery system, nanotherapeutics, nanobots, nanoshells, nanotubes, gene therapy, and vaccination and immunization improvement rank among the functional structures that pharmaceuticals will borrow from nanotechnology while chip technology, quantum dots and sensor nanobots emerge advantageous for diagnostic purposes [31, 32]. However, reconstructive medicine and tissue engineering look forward to applying nanotechnology to cover the gap in clinical practice by providing biodegradable and biocompatible tissues [33]. Radiation enhancers are also under investigation to increase the usability of radiotherapy in war against cancer [34]. Personalized medicine is the other field of interdisciplinary medical science that is enjoying the nanomaterials like theranostics and data management to improve patient care based on certain patient’s needs, genetic and health status [35].

## Concluding remarks

In conclusion, it should be stated that nanopharmaceuticals may pave the way for new therapies for unmet medical needs and optimization of the existing therapies with their promising features. But, in the meanwhile their limitations should also be considered since adverse events may come out during their use in the populations with metabolic variations as well as health status. The nanodrugs modified biokinetics necessitate well established toxicology profiling through in vitro and especially in vivo tests since the lab tests mostly cover their toxicity in cell lines with different physiological properties rather than the healthy people and patients. The test endpoints may include oxidative stress burden, inflammatory system, genotoxicity, carcinogenicity, respiratory, cardiovascular, central and peripheral nervous system, hematopoietic and lymphatic system, developmental and reproductive toxicity. To achieve this goal, validated and standard test protocols as well as reference standards seem critical. Nonetheless, Quality by Design (QbD) approach adopted by the FDA gains importance to manufacture nanodrugs in reliable and reproducible processes. Therefore, it would be advisable for the regulatory authorities to enforce the nanopharmaceutical industries to conduct comprehensive toxicity tests and monitor the adverse drug reaction reports closely to act accordingly and inform the public. Proper collection of possible toxicities and adverse events from the medical and public community is recommended.

## Abbreviations

ADME: Adsorption, Distribution, Metabolism and Excretion
CNS: Central Nervous System
EMA: European Medicine Agency
EPA: Environmental Protection Agency
FDA: Food and Drug Administration
NIOSH: National Institute for Occupational Safety and Health
QbD: Quality by Design
ROS: Reactive Oxygen Species
WHO: World Health Organization
