# Erratum to: Opportunities and obstacles to the development of nanopharmaceuticals for human use

**DOI:** 10.1186/s40199-016-0164-7

**Published:** 2016-11-10

**Authors:** Nasser Nassiri Koopaei, Mohammad Abdollahi

**Affiliations:** 1 Department of Pharmaceutics, School of Pharmacy, University of Florida, Orlando, USA; 2 Department of Toxicology & Pharmacology, Faculty of Pharmacy and Pharmaceutical Sciences Research Center, Tehran University of Medical Sciences, Tehran, Iran; 3 Endocrinology and Metabolism Research Center, Endocrinology and Metabolism Clinical Sciences Institute, Tehran University of Medical Sciences, Tehran, Iran; 4 Toxicology Interest Group, Universal Scientific Education and Research Network, Tehran University of Medical Sciences, Tehran, Iran

## Erratum

After publication of this article [1], the authors clarify that Nasser Nassiri Koopaei was affiliated to “Faculty of Pharmacy and Pharmaceutical Sciences Research Center, Tehran University of Medical Sciences, Tehran, Iran” at the time the research was carried out.
